# The complete chloroplast genome sequence of *Canarium album*

**DOI:** 10.1080/23802359.2019.1674711

**Published:** 2019-10-11

**Authors:** Yi Wang, Jiabo Hao, Bin Lu

**Affiliations:** Laboratory of Forest Plant Cultivation and Utilization, Yunnan Academy of Forestry, Kunming, P.R. China

**Keywords:** *Canarium album*, chloroplast, Illumina sequencing, phylogenetic analysis

## Abstract

The first complete chloroplast genome (cpDNA) sequence of *Canarium album* was determined from Illumina HiSeq pair-end sequencing data in this study. The cpDNA is 163,347 bp in length, contains a large single-copy (LSC) region of 87,838 bp and a small single-copy (SSC) region of 13,935 bp, which were separated by a pair of inverted repeats (IRs) regions of 30,787 bp. The genome contains 129 genes, including 84 protein-coding genes, 8 ribosomal RNA genes, and 37 transfer RNA genes. The overall guanine-cytosine (GC) content of the whole genome is 37.5%, and the corresponding values of the LSC, SSC, and IR regions are 35.8%, 32.6%, and 41.1%, respectively. Further, phylogenomic analysis showed that *C. album* clustered together with *Boswellia sacra*.

*Canarium album* (Chinese olive) is a fruit tree belonging to genus Canarium with the Burseraceae family, and whose natural distribution in the southeast area of China (Kuo et al. 2015). *C. album* has more than two thousand years of cultivation history in China (Wei et al. 2019). *Canarium album* has also been introduced to other Asian tropical and semi-tropical regions (He and Xia 2011). The fruit of *C. album* has relatively low oil contents, and are generally processed in the food industry to beverages, candy, and confections (Ssonko and Xia 2005). Their dried fruits are also a Chinese traditional medicine used for the treatment of faucitis, stomatitis, hepatitis, and toxicosis (Ding 1999). However, there has been no genomic studies on *C. album*.

Herein, we reported and characterized the complete *C. album* plastid genome (MN106250). One *C. album* individual (specimen number: 201805041) was collected from Puwen, Yunnan Province of China (23°76′18″N, 101°28′37″E). The specimen is stored at Yunnan Academy of Forestry Herbarium, Kunming, China, and the accession number is YAFH0012735. DNA was extracted from its fresh leaves using DNA Plantzol Reagent (Invitrogen, Carlsbad, CA).

Paired-end reads were sequenced by using Illumina HiSeq system (Illumina, San Diego, CA). In total, about 30.1 million high-quality clean reads were generated with adaptors trimmed. Aligning, assembly, and annotation were conducted by CLC *de novo* assembler (CLC Bio, Aarhus, Denmark), BLAST, GeSeq (Tillich et al. 2017), and GENEIOUS version 11.0.5 (Biomatters Ltd, Auckland, New Zealand). To confirm the phylogenetic position of *C. album*, other four species of family *Burseraceae* from NCBI were aligned using MAFFT version 7 (Katoh and Standley 2013) and maximum likelihood (ML) bootstrap analysis was conducted using RAxML (Stamatakis 2006); bootstrap probability values were calculated from 1000 replicates. *Pistacia weinmaniifolia* (MF630953) was served as the out-group.

The complete *C. album* plastid genome is a circular DNA molecule with the length of 163,347 bp, contains a large single-copy (LSC) region of 87,838 bp and a small single-copy (SSC) region of 13,935 bp, which were separated by a pair of inverted repeats (IRs) regions of 30,787 bp. The overall guanine-cytosine (GC) content of the whole genome is 37.5%, and the corresponding values of the LSC, SSC, and IR regions are 35.8%, 32.6%, and 41.1%, respectively. The plastid genome contained 129 genes, including 84 protein-coding genes, 8 ribosomal RNA genes, and 37 transfer RNA genes. Phylogenetic analysis showed that *C. album* clustered together with *Boswellia sacra* (Figure 1). The determination of the complete plastid genome sequences provided new molecular data to illuminate the *Burseraceae* evolution.

**Figure 1.**
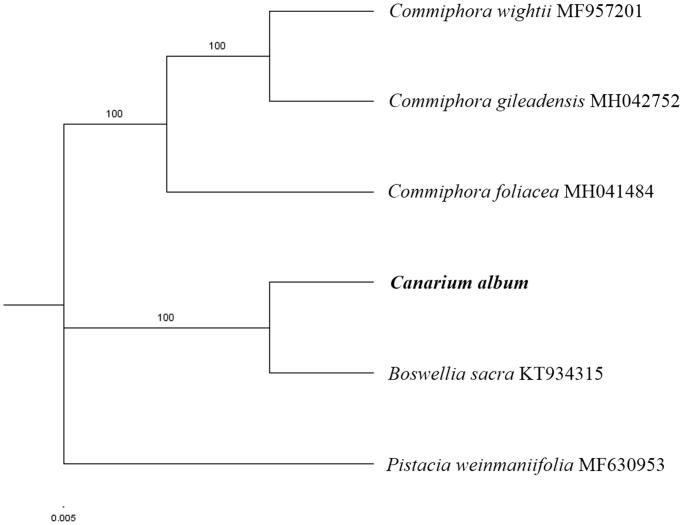
The maximum-likelihood tree based on five chloroplast genomes of *Burseraceae*. The bootstrap value based on 1000 replicates is shown on each node.
